# Progestogen-only oral contraceptives: Number 4 - April 2022

**DOI:** 10.1055/s-0042-1748754

**Published:** 2022-05-27

**Authors:** Rogério Bonassi Machado, Carlos Alberto Politano

**Affiliations:** 1 Faculdade de Medicina de Jundiaí, Jundiaí, SP, Brazil; 2 Universidade Estadual de Campinas, Campinas, SP, Brazil

## Key points

- Progestogen-only oral contraceptives comprise pills composed of progestogens with distinct contraceptive properties, including central or peripheral effects.
- Norethisterone acetate 0.35 mg, desogestrel 75 mcg and drospirenone 4 mg are the progestogen pills available in Brazil. The main mechanism of contraceptive action of desogestrel and drospirenone is the inhibition of the ovulation. The main effect of norethisterone is the alteration of cervical mucus.
- Progestogens used alone for contraception that promote inhibition of the ovulation have greater contraceptive efficacy.
- The bleeding profile of progestogen-only oral contraceptives is regimen dependent. Desogestrel and norethisterone taken continuously have a variable bleeding pattern, ranging from amenorrhea to spotting or even irregular bleeding. Drospirenone alone in a 24/4 regimen has a predictable bleeding pattern in most cases.
- As progestogen pills have a lower risk of cardiovascular events, they are particularly indicated for women with contraindications to combined contraceptives, given the absence of estrogen in their formulations.

## Recommendations

- Progestogen pills containing desogestrel or drospirenone have lower failure rates due to the antiovulatory effect and should be considered for women who require highly effective contraceptives.
- The use of oral desogestrel alone, as well as of norethisterone, should be continuous. Drospirenone alone should be used for 24 days actively followed by a four-day interval.
- All progestogen-only pills are indicated and safe for use in nursing mothers.
- Counseling about the irregular bleeding pattern that may occur when a progestogen pill is prescribed is essential. Prior guidance leads to greater continuity and adherence to this contraceptive modality.
- Progestogen pills may be indicated for obese women, smokers, hypertensive or those with risk factors for cardiovascular disease.
- Progestogen-only oral contraceptives are not associated with a higher risk of venous thromboembolism and may even be indicated for women with a personal history of deep venous thrombosis or pulmonary embolism.
- There is no restriction on the use of progestogen pills by women with a history of cardiovascular disease, including myocardial infarction or stroke.
- Progestogen pills are not associated with reduced bone mineral density.

## Background

Approximately 100 million women worldwide currently use a combined oral contraceptive (COC) containing an estrogen-progestogen combination. 1 Combined oral contraceptives are associated with a higher risk of venous thromboembolism (VTE) and cardiovascular disease. 2 3 The World Health Organization (WHO) attests that progestogen-only oral contraceptives, also known as POPs (progestin-only pills), do not offer a higher risk for VTE, myocardial infarction, and stroke. 4 In this sense, aspects related to safety may result in a greater prescription of POPs for a greater number of women eligible for this contraceptive modality. In Brazil, an analysis of 1,113 women from the supplementary health system showed that the use of a single POP, desogestrel, was reported by 18% of users of contraceptive methods. 5 The most relevant aspects of POPs should be observed in view of the option or medical indication, since all methods containing only progestogens have different biochemical and pharmacological characteristics, in addition to the contraceptive effect itself. The first question that may generate doubt refers to the “progestogen pills” term, which is widespread in the gynecological setting. This group includes compounds previously called minipills, which contained norethisterone, lynestrenol and levonorgestrel (the last two no longer available in Brazil), in addition to desogestrel alone. Recently, drospirenone alone has been incorporated into the group of POPs. 6 The contraceptive mechanism of action is different among POPs. The main mechanism of action of minipills is the alteration of the cervical mucus and secondarily, the endometrial activity hostile to implantation. 7 On the other hand, desogestrel and drospirenone alone have gonadotropic blockade as their main mechanism of action, which offers greater contraceptive efficacy for women who are not breastfeeding. 8 9 Thus, although “progestogen pills” represent the different isolated progestogens used orally, the mechanisms of action are distinct and ultimately, reveal the main characteristic of a contraceptive: effectiveness. The aim of this document is to critically analyze the main characteristics of POPs available in Brazil, with emphasis on frequently asked questions in the practice of professionals involved with the subject.

## What are the main differences between POPs? What are the effectiveness rates of these compounds?

Norethisterone 0.35 mg, desogestrel 75 mcg and drospirenone 4 mg constitute the isolated oral progestogens used in contraception in Brazil. Norethisterone and desogestrel correspond to progestogens structurally related to 19-nortestosterone, synthesized from modi fications in the testosterone molecule. Drospirenone, in turn, is a progestogen structurally related to 17α -spironolactone. 10 Although norethisterone and desogestrel interact with the androgen receptor based on their structural conformation, androgenic effects such as acne and hirsutism are rarely observed clinically. This is explained by the small dose of norethisterone used for contraception (0.35 mg) and the antiovulatory action by suppressing the luteinizing hormone levels of desogestrel at a dose of 75 mcg. The dose of norethisterone used in contraception is insufficient for expressive androgenic stimulation of terminal effectors in the pilosebaceous unit. Desogestrel provides less androgenic activity as a result of gonadotropic blockade and consequent reduction in ovarian testosterone production. 10 Isolated drospirenone, in turn, in addition to gonadotropic blockade, has an antiandrogenic effect, which may promote improvement in acne. 11 Other relevant aspect refers to the way POPs are used. Norethisterone and desogestrel are used continuously, while drospirenone is used in a cyclic regimen, with 24 active pills followed by four days of placebo. 10 11 The effectiveness of POPs depends on the mechanism of action. As norethisterone alone has the main effect on cervical mucus, it depends on high motivation for daily use at the same time (with a maximum delay of three hours). As a result, contraceptive failure rates range from 4 to 7.9 pregnancies per 100 women per year. 12 13 Among POPs that provide gonadotropic blockade, desogestrel alone has a failure rate of 0.14/100 women per year, 14 while drospirenone shows a failure rate of 0.72/100 women per year 15 so, these are classified as highly effective methods.

## How should the optimal use of POPs be and what factors can influence their effectiveness?

The main mechanisms of action of POPs must be considered to obtain greater effectiveness. As norethisterone acts by promoting changes in the cervical mucus, its use demands care: must be continuous and at the same time, with a maximum variation of three hours. Studies show that minipills have a lower failure rate in women over 40 years of age, probably as a result of the natural decline in fertility in this age group. 16 As most women under 40 years of age maintain ovulatory cycles, the effect of norethisterone on cervical mucus should be intense, aiming at greater effectiveness. The effect on cervical mucus, reducing sperm penetration, occurs from 4 to 22 hours after the first dose of progestogen, and the repetition of doses causes difficulty in sperm ascent in the subsequent 24 hours, provided there is no interruption or forgetting of doses. There is no evidence that other factors, such as weight or smoking can interfere with the antisperm activity of small doses of norethisterone. 16 On the other hand, desogestrel and drospirenone alone have an antiovulatory effect, showing greater efficacy regardless of age, and are currently more appropriate choices of a POP. Studies with intentional delays or omission of active pills were performed to attest the antiovulatory effect of progestogens alone. Desogestrel used continuously was evaluated after three 12-hour delays in taking the dose, showing an escape ovulation rate of 1% generally occurring after seven days. 17 Thus, guidance in case of forgetting the dose of this contraceptive formulation is to respect the 12-hour period. Drospirenone alone at a dose of 4 mg is used in a cyclic regimen of 24 active pills, followed by four placebo pills. A study with four intentional 24-hour delays in the administration of active pills showed an escape ovulation rate of 0.8%. 18 The difference can be explained by the properties of drospirenone, which has plasma half-life of approximately 33 hours. In fact, mean rates of escape ovulation with combined oral contraceptives are around 2%. 19 Factors that could negatively influence the antiovulatory activity of drospirenone and desogestrel alone, such as obesity or smoking, have not been identified. Drug interactions, particularly with anticonvulsants, should be considered. 20

## What are the effects of progestogens alone on lactation?

The importance of contraception in the puerperal period is widely known, and the use of effective contraceptive methods is recommended as early as possible. Most contraceptives, except for combined hormonal contraceptives, can be indicated in the immediate postpartum period both for lactating and non-lactating women. 20 Progestogens alone are traditionally indicated in contraception for breastfeeding women, as they do not present adverse effects on lactation. 21 Classically, the WHO recommends starting progestogens from the sixth week after delivery for breastfeeding women, and it can be started immediately by non-lactating women. 22 However, for patients at high cardiovascular or thromboembolic risk, progestogens can be prescribed in the immediate postpartum period, even for breastfeeding women, since the method does not add risk of thrombosis. 23 Furthermore, desogestrel doses higher than 75 mcg do not cause any difference in the composition or amount of milk, nor in the development and growth of children, compared to women who used postpartum copper IUDs. 24 An important aspect discussed recently, during the pandemic, refers to the clotting disorder involving the infection by SARSCoV-2. 25 As puerperal women can be contaminated and the hormonal condition of this period itself increases the thromboembolic risk, concerns about the contraceptive method used have been considered, reinforcing the indication of methods containing progestogen-only. 26 Drospirenone alone at a dose of 4 mg was evaluated in a subgroup of lactating women, calculating the passage of the hormone into maternal milk in 24 hours and the consequent exposure of the newborn to drospirenone. Considering a daily intake of 800 mL of breast milk, in which the drospirenone concentration reached 4478 ng, 0.11% of the progestogen was transferred to the newborn, attesting the safety of the method during breast-feeding. Thus, drospirenone can also be indicated in the postpartum period to lactating women. 27

## How is the bleeding profile characterized with the different progestogens in contraception?

The bleeding pattern with the use of POPs is variable, ranging from amenorrhea to frequent and irregular bleeding. In general, women exposed to minipills such as norethisterone will continue to ovulate and have regular cycles, while those who experience ovarian suppression will have irregular and unpredictable bleeding. The mechanisms involved in bleeding during the use of progestogen-only pills are not well established. Possible explanations for the bleeding alterations when using contraceptives include more changes in tissue perfusion in combination with local angiogenic factors, together with the permeability of superficial vessels and the change of receptor functions for endometrial steroid hormones. 28 In fact, irregular bleeding is the most commonly cited reason for discontinuing POPs, and occurs in up to 25% of users. 14 29 The WHO recommends using the analysis by “reference period” (RP), 30 defined as periods of time measured in number of days to analyze the bleeding pattern with different contraceptives. In most current studies, the 90-day RP is used to characterize the bleeding pattern with hormonal contraceptives, particularly with progestogens alone. In a one year follow-up study, two progestogen-only contraceptives, desogestrel 75 mcg and levonorgestrel 30 mcg, were compared. 14 The analysis of the bleeding pattern followed the WHO nomenclature. In the RP, about 50% of women on desogestrel 75 mcg experienced amenorrhea or infrequent bleeding compared to 10% of levonorgestrel users. Only 4% of women on desogestrel experienced frequent bleeding, in contrast to 10% of women in the levonorgestrel group. The incidence of prolonged bleeding decreased with time in both treatment groups. As the regimen of drospirenone alone has a four-day break between active pills, the bleeding profile is distinct, and evaluated in 30-day RPs. In a phase 3 double-blind, randomized, controlled clinical trial study involving healthy women aged 18-45 years, the bleeding profile of the contraceptive containing drospirenone only (4 mg) on a 24/4 cyclic regimen was compared to the contraceptive containing desogestrel (75 mcg) used continuously over nine cycles. 31 Scheduled bleeding was defined as any bleeding or spotting that occurred during the hormone-free intervals (between days 25 and 28, ± one day), lasting up to eight consecutive days for women using drospirenone. For this group, unscheduled bleeding was defined as any blood loss or spotting occurring between days 2 and 23 of each cycle, corresponding to the period of intake of active pills. For the group of desogestrel users, definitions for scheduled bleeding were not considered, and all days of bleeding or spotting during the use of active pills were recorded. Women who used desogestrel experienced a percentage decrease in bleeding/spotting rates from 74% to 45.3% between cycles 2 and 9, respectively. When considering only unscheduled bleeding/spotting, the percentage for drospirenone users was significantly lower compared to desogestrel users, particularly between cycles 2-6 and 2-9. The mean number of days of unscheduled bleeding during cycles 2-9 was significantly lower for drospirenone (21.5 days) compared to desogestrel (34.7 days). There was also a trend towards fewer bleeding/spotting days over time for drospirenone users (mean 13.1 days between cycles 2-4 to 9.7 days between cycles 7-9). The desogestrel group experienced a mean reduction from 16.9 days to 10.8 days, between cycles 2-4 and 7-9, respectively. The number of bleeding/spotting days was lower in the group of women who used drospirenone at all defined treatment periods.

## What is the impact of progestogen only contraceptives on bone mass?

The effects of estrogens as antiresorptive agents on bone mass are widely known. 32 Low estrogen levels are associated with inadequate bone remodeling, with increased bone resorption activity. 33 The use of estrogen-free contraceptives with antigonadotropic effects may raise doubts about bone metabolism. Studies show that mean estradiol concentrations in users of intramuscular depot progestagen (medroxyprogesterone acetate – MPA) are lower than those observed with use of oral desogestrel or drospirenone. In fact, estradiol rates measured with the use of MPA, desogestrel 75 mcg and drospirenone 4 mg, were 26.6 pg/mL, 34 54.4 pg/mL 35 and 48.4 pg/mL, 36 respectively. Note that according to the hierarchy of tissue-specific estrogenic response, levels below 20 pg/mL are associated with substantial bone loss. 37 Studies on the subject are focused especially on the use of intramuscular depot MPA, due to the pronounced gonadotropic blockade and the consequent hypoestrogenic effect. In a systematic review, Curtis et al. 38 observed that a study reported a higher number of stress fractures in users of MPA compared to non-users of the contraceptive. However, this finding was not significant after checking baseline BMD in both groups. The authors also found that cross-sectional studies demonstrate a decrease in BMD with variations within one standard deviation for women who used MPA versus non-users and, according to longitudinal studies, there was a recovery of BMD after discontinuation of use. Thus, they concluded that, except for depot MPA, other progestogen-only contraceptives would not affect BMD.

## What is the risk of having cardiovascular disease with progestogen-only contraceptives?

The association between POPs and the risk of various cardiovascular outcomes, including VTE, myocardial infarction, stroke, hypertension, and diabetes by route of administration was also studied. In a review of 19 studies 39 based on the random effects model, the pooled adjusted relative risks (RRs) for VTE, myocardial infarction and stroke for POP users versus non-users were 1.06 (95% confidence interval [CI]: 0.70-1.62), 0.98 (95%CI: 0.66-1.47) and 1.02 (95% CI: 0.72-1.44), respectively. No effect of POP use on blood pressure was found. Hence the assumption that the oral use of POPs is not associated with a higher risk of developing various cardiometabolic outcomes. On the other hand, women with past medical conditions that offer higher risk of thrombosis should also not use estrogen-containing contraceptives. Little is known about POPs in this situation. In the systematic review by Tepper et al., 40 most evidence do not suggest a higher risk of venous or arterial events with the use of POPs. Thus, hypertensive or smoker women can still benefit from using POPs to prevent pregnancy.

## According to eligibility criteria, under which conditions should oral progestogen-only methods be prioritized?

Progestogen-only methods have few contraindications. They are also highly indicated in clinical situations in which there is an absolute contraindication to the use of hormonal contraceptives containing estrogens (combined hormonal contraceptives). The unrestricted indications (categories 1 and 2) for progestogen-only methods, when estrogen-associated methods are generally contraindicated, are summarized in chart 1. 22

**Chart 1.** Indications for progestogen-only methods, particularly when the use of combined hormonal contraceptives is contraindicated

Patient choice, regardless of age Lactation, including the period from 6 weeks to 6 months after delivery Smokers aged over 35 years Obesity (BMI > 30 kg/m ^2^ ) Multiple risk factors for cardiovascular disease Controlled high blood pressure or levels of 140-159/90-99 mmHg Known thrombophilia, history of DVT or PTE, thromboembolism on anticoagulant use Major surgeries with immobilization Dyslipidemia Valvular heart disease, even complicated Systemic lupus erythematosus, except in the presence of antiphospholipid antibodies or severe thrombocytopenia Headaches, including migraine with aura Epilepsy, depression Menstrual irregularities, endometriosis, benign ovarian cysts, cervical ectopia, gestational trophoblastic neoplasia, cervical intraepithelial neoplasia Benign breast diseases Endometrial and ovarian cancer Uterine fibroids, pelvic inflammatory disease, STIs High risk for HIV, HIV positive, AIDS Diabetes mellitus Thyroid diseases Gallbladder diseases, hepatitis, compensated cirrhosis, benign liver tumors Anemias (including thalassemia and sickle cell anemia) Concomitant use of antiretrovirals, antifungals, broad-spectrum antibiotics, and antiparasitics

BMI: body mass index; DVT: deep vein thrombosis; PTE: pulmonary thromboembolism; STIs: sexually transmitted infections; AIDS: acquired immunodeficiency syndrome; HIV: human immunodeficiency virus

Source: World Health Organization. Medical eligibility criteria for contraceptive use [Internet]. 5th ed. Geneva: WHO; 2015 [cited 2021 Oct 24]. Available from: https://www.who.int/publications/i/item/9789241549158 . 22

## Final considerations

The class of progestogen-only oral contraceptives combine efficacy with very broad indications, including critical clinical situations, particularly when combined hormonal methods are not recommended. Although they are described together, progestogen contraceptive methods must be analyzed individually. The specific characteristics of each compound require a critical analysis focused on the clinical condition where the method is intended to be instituted. Note that the unpredictable bleeding pattern is the main element responsible for the abandonment of POPs. In this sense, desogestrel and drospirenone alone are different; drospirenone showed better cycle control in phase 3 studies, allowing greater contraceptive coverage in situations where estrogen is contraindicated.

National Specialized Commission on Contraception of the Brazilian Federation of Gynecology and Obstetrics Associations (FEBRASGO)

President:

Rogerio Bonassi Machado

Vice-President:

Ilza Maria Urbano Monteiro

Secretary:

Jaqueline Neves Lubianca

Members:

Carlos Alberto Politano

Cristina Aparecida Falbo Guazzelli

Edson Santos Ferreira Filho

Jarbas Magalhães

Luis Carlos Sakamoto

Maria Auxiliadora Budib

Mariane Nunes de Nadai

Milena Bastos Brito

Sheldon Rodrigo Botogoski

Tereza Maria Pereira Fontes

Valeria Barbosa Pontes

Zsuzsanna Ilona Katalin de Jarmy Di Bella
